# Prehospital stroke detection scales: A head-to-head comparison of 7 scales in patients with suspected stroke

**DOI:** 10.1177/17474930241275123

**Published:** 2024-09-10

**Authors:** Luuk Dekker, Walid Moudrous, Jasper D Daems, Ewout FH Buist, Esmee Venema, Marcel DJ Durieux, Erik W van Zwet, Els LLM de Schryver, Loet MH Kloos, Karlijn F de Laat, Leo AM Aerden, Diederik WJ Dippel, Henk Kerkhoff, Ido R van den Wijngaard, Marieke JH Wermer, Bob Roozenbeek, Nyika D Kruyt

**Affiliations:** 1 Department of Neurology, Leiden University Medical Center, Leiden, The Netherlands; 2 Department of Neurology, Maasstad Hospital, Rotterdam, The Netherlands; 3 Department of Neurology, Erasmus MC University Medical Center, Rotterdam, The Netherlands; 4 Department of Public Health, Erasmus MC University Medical Center, Rotterdam, The Netherlands; 5 Amsterdam University Medical Center, Amsterdam, T﻿he Netherlands; 6 Department of Emergency Medicine, Erasmus MC University Medical Center, Rotterdam, The Netherlands; 7 Emergency Medical Services Hollands-Midden, Leiden, The Netherlands; 8 Department of Biomedical Data Sciences, Leiden University Medical Center, Leiden, The Netherlands; 9 Department of Neurology, Alrijne Hospital, Leiderdorp, The Netherlands; 10 Department of Neurology, Groene Hart Hospital, Gouda, The Netherlands; 11 Department of Neurology, Haga Hospital, The Hague, The Netherlands; 12 Department of Neurology, Reinier de Graaf Gasthuis Hospital, Delft, The Netherlands; 13 Department of Neurology, Albert Schweitzer Hospital, Dordrecht, The Netherlands; 14 Department of Neurology, Haaglanden Medical Center, The Hague, The Netherlands; 15 University NeuroVascular Center (UNVC), Leiden-The Hague, The Netherlands; 16 Department of Neurology, University Medical Center Groningen, Groningen, The Netherlands

**Keywords:** Cerebrovascular diseases, stroke, ischemic stroke, hemorrhagic stroke, stroke mimic, triage, paramedic

## Abstract

**Background:**

Several prehospital scales have been designed to aid paramedics in identifying stroke patients in the ambulance setting. However, external validation and comparison of these scales are largely lacking.

**Aims:**

To compare all published prehospital stroke detection scales in a large cohort of unselected stroke code patients.

**Methods:**

We conducted a systematic literature search to identify all stroke detection scales. Scales were reconstructed with prehospital acquired data from two observational cohort studies: the Leiden Prehospital Stroke Study (LPSS) and PREhospital triage of patients with suspected STrOke (PRESTO) study. These included stroke code patients from four ambulance regions in the Netherlands, including 15 hospitals and serving 4 million people. For each scale, we calculated the accuracy, sensitivity, and specificity for a diagnosis of stroke (ischemic, hemorrhagic, or transient ischemic attack (TIA)). Moreover, we assessed the proportion of stroke patients who received reperfusion treatment with intravenous thrombolysis or endovascular thrombectomy that would have been missed by each scale.

**Results:**

We identified 14 scales, of which 7 (CPSS, FAST, LAPSS, MASS, MedPACS, OPSS, and sNIHSS-EMS) could be reconstructed. Of 3317 included stroke code patients, 2240 (67.5%) had a stroke (1528 ischemic, 242 hemorrhagic, 470 TIA) and 1077 (32.5%) a stroke mimic. Of ischemic stroke patients, 715 (46.8%) received reperfusion treatment. Accuracies ranged from 0.60 (LAPSS) to 0.66 (MedPACS, OPSS, and sNIHSS-EMS), sensitivities from 66% (LAPSS) to 84% (MedPACS and sNIHSS-EMS), and specificities from 28% (sNIHSS-EMS) to 49% (LAPSS). MedPACS, OPSS, and sNIHSS-EMS missed the fewest reperfusion-treated patients (10.3–11.2%), whereas LAPSS missed the most (25.5%).

**Conclusions:**

Prehospital stroke detection scales generally exhibited high sensitivity but low specificity. While LAPSS performed the poorest, MedPACS, sNIHSS-EMS, and OPSS demonstrated the highest accuracy and missed the fewest reperfusion-treated stroke patients. Use of the most accurate scale could reduce unnecessary stroke code activations for patients with a stroke mimic by almost a third, but at the cost of missing 16% of strokes and 10% of patients who received reperfusion treatment.

## Introduction

Reperfusion treatment with intravenous thrombolysis (IVT) or endovascular thrombectomy (EVT) is highly effective in ischemic stroke, but the effects of both are time sensitive.^1,2^ Stroke code patients, that is, patients suspected of having an acute stroke by emergency medical services (EMS) paramedics, are therefore transported to the nearest stroke center with a pre-notification ensuring that a stroke team is standing by at the emergency department (ED) and a computed tomography (CT) scanner is available.^3,4^ However, up to 50% of stroke code patients are diagnosed with a stroke mimic instead of a stroke, including functional neurological disorders, peripheral vestibular disorders, or epileptic seizures.^5,6^ These stroke mimics often do not require urgent presentation in a specialized stroke center, while placing considerable strain on EMS and ED resources and imposing substantial additional costs.^7^ Furthermore, acute stroke code presentations are associated with unnecessary neuroimaging tests and the inappropriate administration of IVT.^5–8^

Accurate stroke triage in the field is therefore crucial. To this end, several prehospital stroke detection scales are used by EMS paramedics.^9–22^ However, studies that investigated these scales often had retrospective designs, were based on small or selected patients cohorts, or do not reflect EMS practices as assessments were conducted by physicians at the ED rather than by paramedics in the prehospital setting.^23–29^ Moreover, some scales lack external validation, and previous head-to-head comparisons only included a limited subset of these scales.^30–36^ These methodological shortcomings contribute to inconsistent findings in comparative studies and systematic reviews, impeding guideline recommendations for the preferred use of one scale over the others.^3,4,37–39^ Finally, although these scales are designed to detect stroke patients in general, from a practical point of view it is most important not to overlook patients eligible for reperfusion therapy.

## Aims

We aimed to (1) systematically identify all published prehospital stroke detection scales and (2) to compare their diagnostic performance for detecting stroke patients (ischemic, hemorrhagic, or transient ischemic attack (TIA)); and ischemic stroke patients eligible for reperfusion treatment.

## Method

### Identification of scales

To identify all published stroke detection scales, we used a Cochrane review that included studies until 2018.^37^ We performed an additional literature search based on the search strategy and findings of that review in PubMed and EMBASE up to May 2023 (Supplemental Appendix S1). Two reviewers (L.D. and E.F.H.B.) independently screened titles and abstracts for eligibility and obtained full-text versions from all English studies considered relevant. We included ordinal scales that utilized clinical or demographic characteristics with a specified cut-point value for stroke detection. Scales originally designed for in-hospital use were included if prehospital use was considered feasible. We excluded scales specifically designed to identify distinct stroke subtypes (e.g. large-vessel occlusion, posterior circulation, or hemorrhagic stroke), to identify only patients eligible for reperfusion treatment, or to assess stroke severity or prognosis, and scales using resources not routinely available in the prehospital setting (e.g. telecommunication).

### Study population and data collection

We used individual patient data from two large, prospective observational cohort studies: the Leiden Prehospital Stroke Study (LPSS) and the PREhospital triage of patients with suspected STrOke (PRESTO) study.^40,41^ These studies enrolled stroke code patients from four EMS regions in the Netherlands, serving approximately 4 million inhabitants. These regions encompassed 15 stroke centers, all providing 24/7 evaluation of stroke code patients with a stroke team, CT and CT-angiography availability, and IVT administration. Of these centers, 10 were primary stroke centers, 2 were thrombectomy-capable stroke centers, and 3 were comprehensive stroke centers with additional neurosurgical capacities. All patients aged ⩾18 years for whom EMS paramedics activated an acute stroke code between July 2018 and October 2019 were included. Standard practice was to activate a stroke code if there was a suspicion of acute stroke, based on a positive Face-Arm-Speech-Time (FAST) test or other neurological symptoms at the discretion of the individual paramedic.^9^ At the time, policy was to transport a stroke code patient to the nearest stroke center. Paramedics routinely recorded patient characteristics such as time of symptom onset, blood pressure, and blood glucose in electronic transport records. In addition, paramedics documented 9 (PRESTO) to 11 (LPSS) neurological observations in an application prior to hospital arrival. Electronic hospital records were used to extract demographic characteristics, medical history, medication use, stroke severity with the National Institutes of Health Stroke Scale (NIHSS) assessed by physicians upon presentation at the ED,^42^ neuroimaging findings, reperfusion treatment with IVT and EVT, and the final diagnosis as determined by the treating physician either upon discharge (PRESTO) or after 3 months (LPSS).

LPSS and PRESTO were approved by the relevant Medical Ethical Review Committees and the Institutional Review Boards of all participating centers. A waiver for consent was granted for both studies. Our reporting adhered to the Standards for the Reporting of Diagnostic Accuracy Studies (STARD) guidelines.^43^

### Scale reconstruction and missing data

For each patient, scales were reconstructed with the prehospital neurological observations documented by paramedics. Scales that required items that were not available from the prehospital assessments were excluded. Multiple Imputation by Chained Equations (MICE) with five imputations was used for missing prehospital observations (Supplemental Appendix S2).

### Statistical analysis

The diagnostic performance of scales was assessed by calculating the accuracy for a final diagnosis of stroke at the specified cut-point from the original studies, as well as the sensitivity, specificity, positive predictive value (PPV), and negative predictive value (NPV) with corresponding 95% Clopper–Pearson confidence intervals (CIs). Stroke was defined as ischemic stroke, intracranial hemorrhage, or TIA. Scale accuracies were compared with the Wald test. Furthermore, we assessed the proportion of missed reperfusion-treated stroke patients, defined as the number of IVT- or EVT-treated ischemic stroke patients that were missed by each scale divided by the total number of ischemic stroke patients that received reperfusion treatment (i.e. excluding IVT-treated patients diagnosed with a stroke mimic). We negated possible eligibility criteria of scales as stated in the original studies in order to evaluate all scales in the same patient cohort to ensure a fair head-to-head comparison.

We performed three sensitivity analyses. First, to assess the validity of our primary analysis with MICE, we compared these results with the results obtained by replacing missing prehospital observations with findings from corresponding items of the NIHSS at the ED. Second, we performed a complete case analysis, excluding patients with one or more missing prehospital observations required for the reconstruction of a scale. Third, to specifically assess scales’ diagnostic performance for patients with ischemic or hemorrhagic stroke, we repeated the primary analysis after excluding patients with a TIA.

A two-sided *p*-value ⩽ 0.05 was considered statistically significant. MICE was performed in R (version 4.1.2) with the MICE package (version 3.14.0), and other analyses with SPSS (version 28.0).

## Results

### Systematic literature search

We identified 14 stroke detection scales that met our criteria: the Balance, Eyes, Face-Arm-Speech-Time (BE-FAST),^10^ Cincinnati Prehospital Stroke Scale (CPSS),^11^ Clinical Information, Vital signs, and Initial Labs—Age, Stroke risks, history of Seizure or psychiatric disease, Sugar level, Asymmetry, not Ambulating, blood Pressure (CIVIL-ASAP),^12^ Face-Arm-Speech-Time (FAST),^9^ Finnish Prehospital Stroke Scale (FPSS),^13^ Guangzhou Stroke Scale (GZSS),^14^ Los Angeles Prehospital Stroke Screen (LAPSS),^15^ Melbourne Ambulance Stroke Screen (MASS),^16^ Medic Prehospital Assessment for Code Stroke (MedPACS),^17^ Ontario Prehospital Stroke Screen (OPSS),^18^ PreHospital Ambulance Stroke Test (PreHAST),^19^ Recognition Of Stroke In the Emergency Room (ROSIER),^20^ Staring-Hypertension-atrIal fibrillation-sPeech-weakneSs (SHIPS),^21^ and shortened NIHSS for Emergency Medical Services (sNIHSS-EMS).^22^ All scales had a cut-point of 1 for stroke detection, except for GZSS (⩾2 points) and SHIPS (⩾3 points). The search strategy, PRISMA flowchart of the literature search, and an overview of all scales and their included items can be found in Supplemental Appendix S1, Supplemental Figure S1, and Supplemental Table S1.

### Study population

**Figure 1.**
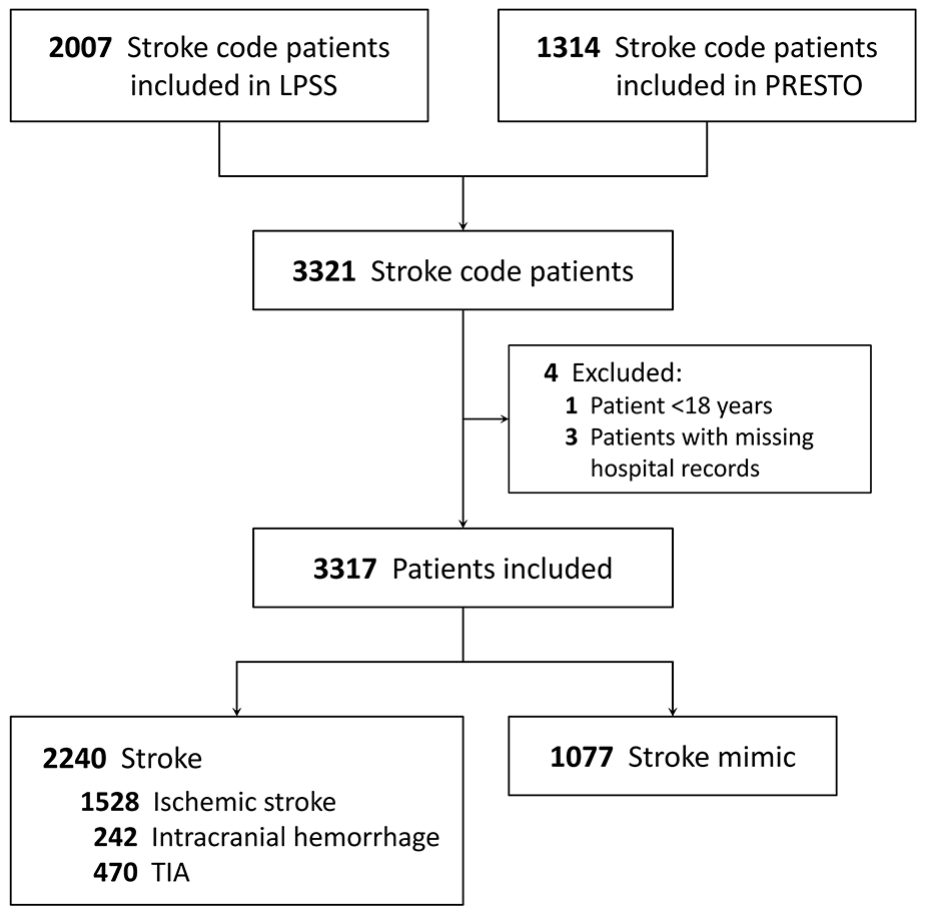
Flowchart of included patients. LPSS: Leiden Prehospital Stroke Study; PRESTO: PREhospital triage in patients with suspected STrOke; TIA: transient ischemic attack.

Of 3321 stroke code patients included in LPSS (*n* = 2007) and PRESTO (*n* = 1314), one was younger than 18 years and three were excluded because of missing hospital records. Of 3317 included stroke code patients, 2240 (67.5%) had a stroke (1528 ischemic, 242 intracranial hemorrhage, and 470 TIA) and 1077 (32.5%) a stroke mimic (Figure 1). Mean age was 71 years and 1606 (48.4%) were women. The median time from stroke onset to ambulance arrival on-site was 82 min (interquartile range (IQR) = 29–254) and from onset to hospital presentation was 111 min (IQR 60–278). The median NIHSS score at the ED was 2 (IQR = 0–6), with 28% of patients having no deficits on the NIHSS. In total, 669 (20.2%) stroke code patients (621 ischemic stroke patients and 48 patients with a stroke mimic) received IVT and 202 (6.1%) patients underwent EVT (Table 1). The most common diagnoses in patients with a stroke mimic were functional neurological disorders (17%), epileptic seizures (14%) and peripheral vestibular disorders (14%).

**Table 1.** Characteristics of stroke code patients.

Characteristic	Total cohort (*n* = 3317)	Stroke patients (*n* = 2240)	Stroke mimic patients (*n* = 1077)
Demographic			
Age (years), mean (SD)	71.0 (14.5)	73.0 (13.1)	66.8 (16.3)
Female sex, *n* (%)	1606 (48.4)	1016 (45.4)	590 (54.8)
Pre-stroke modified Rankin Scale score (range 0–5), median (IQR)	0 (0–1)	0 (0–2)	0 (0–1)
History and medication, *n* (%)			
Ischemic stroke/TIA	973/3297 (29.5)	644/2227 (28.9)	329/1070 (30.7)
Intracerebral hemorrhage	101/3308 (3.1)	54/2234 (2.4)	47/1074 (4.4)
Atrial fibrillation	504/3295 (15.3)	373/2228 (16.7)	131/1067 (12.3)
Myocardial infarction	371/3288 (11.3)	267/2220 (12.0)	104/1068 (9.7)
Diabetes mellitus	687/3296 (20.8)	483/2227 (21.7)	204/1069 (19.1)
Hypercholesterolemia	1748/3293 (53.1)	1229/2224 (55.3)	519/1069 (48.6)
Hypertension	2030/3299 (61.5)	1457/2229 (65.4)	573/1070 (53.6)
Epilepsy (LPSS only)	106/2006 (5.3)	37/1254 (3.0)	69/752 (9.2)
Antiplatelets	1188/3285 (36.2)	826/2222 (37.2)	362/1063 (34.1)
Oral anticoagulation	558/3271 (17.1)	397/2212 (17.9)	161/1059 (15.2)
Prehospital assessment			
Onset-to-EMS arrival time (min), median (IQR)	82 (29–254)	83 (28–276)	80 (32–204)
Systolic blood pressure (mmHg), median (IQR)	160 (142–182)	164 (145–186)	153 (134–174)
Diastolic blood pressure (mmHg), median (IQR)	90 (80–102)	92 (81–105)	88 (77–100)
Blood glucose (mmol/L), median (IQR)	6.5 (5.6–8.0)	6.5 (5.6–8.0)	6.5 (5.6–7.9)
In-hospital assessment			
Onset-to-door time (min), median (IQR)	111 (60–278)	112 (59–307)	110 (64–228)
Total NIHSS score (range, 0–42), median (IQR)	2 (0–6)	3 (1–7)	1 (0–3)
Treatment			
IVT and/or EVT, *n* (%)	763 (23.0)	715 (31.9)	48 (4.5)
IVT, *n* (%)	669 (20.2)	621 (27.7)	48 (4.5)
Door-to-needle-time (min), median (IQR)	21 (16–30)	21 (16–29)	24 (18–33)
EVT, *n* (%)	202 (6.1)	202 (9.0)	0
Door-to-groin-time (min), median (IQR)	53 (29–69)	53 (29-69)	–
Transfer from PSC to CSC for EVT, *n* (%)	75/202 (37.1)	75/202 (37.1)	–

CSC: comprehensive stroke center; EVT: endovascular thrombectomy; IQR: interquartile range; IVT: intravenous thrombolysis; LPSS: Leiden Prehospital Stroke Study; NIHSS: National Institutes of Health Stroke Scale; PSC: primary stroke center; SD: standard deviation; TIA: transient ischemic attack.

### Scale reconstruction

Of the 14 identified scales, seven (CPSS, FAST, LAPSS, MASS, MedPACS, OPSS, and sNIHSS-EMS) could be reconstructed with the available prehospital observations. The other seven scales could not be reconstructed, because no data concerning visual field defects (required for BE-FAST, FPSS, GZSS, PreHAST, and ROSIER), gait imbalance (for BE-FAST and CIVIL-ASAP), asymmetry, history of seizure, epilepsy or psychiatry, and stroke risks (for CIVIL-ASAP), vertigo and Glasgow Coma Scale (for GZSS), on-site loss of consciousness, syncope, or seizure activity (for ROSIER), or history of hypertension or atrial fibrillation (for SHIPS) was documented by paramedics. Because CPSS and FAST use the same clinical items, except for a specific sentence to be repeated for CPSS, these scales were combined in the analyses (CPSS/FAST). An overview of the included scales and their items can be found in Table 2.

**Table 2.** Overview of included prehospital stroke detection scales.

Stroke scale	CPSS/FAST^a^	LAPSS	MASS	OPSS	Med PACS	sNIHSS-EMS
Prehospital observation						
Facial droop	1	1	1	1	1	0–3
Arm motor function	1	1	1	1	1	0–4
Speech disturbance	1	–	1	1	1	0–3
Grip strength	–	1	1	–	–	–
Leg motor function	–	–	–	1	1	0–4
Gaze deviation	–	–	–	–	1	–
Commands	–	–	–	–	–	–
Sensory deficits	–	–	–	–	–	0–2
Consciousness	–	–	–	–	–	0–3
Cut-point	⩾1	⩾1	⩾1	⩾1	⩾1	⩾1^b^

CPSS: Cincinnati Prehospital Stroke Scale; FAST: Face-Arm-Speech-Time test; LAPSS: Los Angeles Prehospital Stroke Screen; MASS: Melbourne Ambulance Stroke Screen; MedPACS: Medic Prehospital Assessment for Code Stroke; OPSS: Ontario Prehospital Stroke Screen; sNIHSS-EMS: shortened National Institutes of Health Stroke Scale for Emergency Medical Services.

a CPSS and FAST use the same clinical items, except for a specific sentence to be repeated for CPSS, and were therefore combined.

b sNIHSS-EMS also aims to specifically detect ischemic stroke patients with an underlying large-vessel occlusion in the anterior circulation using an alternative cut-point of ⩾6 points.

### Missing data

In total, 16.7% of prehospital observations was missing and imputed with MICE for the primary analysis (Supplemental Table S2). This mainly constituted of examinations of sensory deficits (*n* = 1797) and consciousness (*n* = 1317), as these were not routinely documented in PRESTO. Of the remaining observations, 8.1% was missing. Age, sex, pre-stroke modified Rankin Scale score, medical history, medication use, wake-up stroke, onset-to-door time, prehospital and in-hospital blood pressure, blood glucose, all individual prehospital observations and NIHSS items, final diagnosis, and study inclusion were used in the MICE model. Further specification of the imputation method can be found in Supplemental Appendix S2.

### Head-to-head comparison

Table 3 shows the diagnostic performance of each scale for the detection of stroke patients. Accuracies ranged from 0.60 for LAPSS to 0.66 for MedPACS, OPSS, and sNIHSS-EMS. The accuracy of LAPSS (0.60, 95% CI = 0.59–0.62) was significantly lower than of all other scales (*p* < 0.01). In addition, MedPACS, sNIHSS-EMS, and OPSS had significantly higher accuracies than CPSS/FAST and MASS (all *p* ⩽ 0.02) (Table 4). Sensitivity was high for most scales, ranging from 80% (95% CI = 78–81%) for CPSS/FAST to 84% for MedPACS and sNIHSS-EMS (95% CI = 82–85% and 83–86%, respectively), except for LAPSS (66%, 95% CI = 64–68%). Conversely, specificity was generally low (range = 28–34%), except for LAPSS (49%, 95% CI = 46–52%). Moreover, LAPSS had the highest PPV (73%, 95% CI = 71–75%), while MedPACS and OPSS had the highest NPV (both 47%, 95% CI = 43–50%). NPVs of other scales ranged from 41% to 46%. Notably, the proportion of missed reperfusion-treated stroke patients varied from 10.3% for MedPACS to 25.5% for LAPSS.

**Table 3.** Diagnostic performance of prehospital stroke detection scales.

Stroke scale	Accuracy (95% CI)	Sensitivity (95% CI)	Specificity (95% CI)	PPV (95% CI)	NPV (95% CI)	Proportion of missed reperfusion treatments, % (n)		
						Overall (*n* = 715)	IVT (*n* = 621)	EVT (*n* = 202)
MedPACS	0.66 (0.65–0.68)	84% (82–85)	30% (27–33)	71% (70–73)	47% (43–50)	10.3% (74)	11.6% (72)	1.0% (2)
OPSS	0.66 (0.64–0.68)	83% (81–85)	31% (28–34)	71% (70–73)	47% (43–50)	11.2% (80)	12.6% (78)	1.5% (3)
sNIHSS-EMS	0.66 (0.64–0.68)	84% (83–86)	28% (25–31)	71% (69–73)	46% (42–50)	10.5% (75)	11.8% (73)	1.0% (2)
MASS	0.65 (0.63–0.67)	82% (80–84)	30% (27–33)	71% (69–73)	44% (41–48)	13.3% (95)	15.0% (93)	1.0% (2)
CPSS/FAST^a^	0.65 (0.63–0.66)	80% (78–81)	34% (31–37)	72% (70–73)	44% (41–48)	14.8% (106)	16.7% (104)	1.5% (3)
LAPSS	0.60 (0.59-0.62)	66% (64–68)	49% (46–52)	73% (71–75)	41% (38–44)	25.5% (182)	28.0% (174)	5.9% (12)

CI: confidence interval; CPSS: Cincinnati Prehospital Stroke Scale; EVT: endovascular thrombectomy; FAST: Face-Arm-Speech-Time test; IVT: intravenous thrombolysis; LAPSS: Los Angeles Prehospital Stroke Screen; MASS: Melbourne Ambulance Stroke Screen; MedPACS: Medic Prehospital Assessment for Code Stroke; OPSS: Ontario Prehospital Stroke Screen; PPV: positive predictive value; NPV: negative predictive value; sNIHSS-EMS: shortened National Institutes of Health Stroke Scale for Emergency Medical Services.

a CPSS and FAST use the same clinical items and were therefore combined in the analysis (CPSS/FAST).

**Table 4.** Comparison of accuracies of prehospital stroke detection scales.

Stroke scale	Accuracy (95% CI)	LAPSS	CPSS/FAST	MASS	sNIHSS-EMS	OPSS
**LAPSS**	0.60 (0.59–0.62)	X				
**CPSS/FAST** ^a^	0.65 (0.63–0.66)	**<0.01**	x			
**MASS**	0.65 (0.63–0.67)	**<0.01**	0.48	x		
**sNIHSS-EMS**	0.66 (0.64–0.68)	**<0.01**	**<0.01**	**0.02**	x	
**OPSS**	0.66 (0.64–0.68)	**<0.01**	**<0.01**	**<0.01**	0.90	x
**MedPACS**	0.66 (0.65–0.68)	**<0.01**	**<0.01**	**<0.01**	0.89	0.55

Diagnostic accuracies of the scales were compared with the Wald test. Two-sided *p*-values ⩽ 0.05 are shown in bold.

CI: confidence interval; CPSS: Cincinnati Prehospital Stroke Scale; FAST: Face-Arm-Speech-Time test; LAPSS: Los Angeles Prehospital Stroke Screen; MASS: Melbourne Ambulance Stroke Screen; MedPACS: Medic Prehospital Assessment for Code Stroke; OPSS: Ontario Prehospital Stroke Screen; sNIHSS-EMS: shortened National Institutes of Health Stroke Scale for Emergency Medical Services.

a CPSS and FAST use the same clinical items and were therefore combined in the analysis (CPSS/FAST).

### Sensitivity analyses

Replacing missing prehospital observations with findings from corresponding items of the NIHSS at the ED (*n* = 3016) yielded nearly identical results as the primary analysis with MICE (Supplemental Table S3). In the complete case analysis (*n* = 1133), after excluding all patients with one or more missing observations, sensitivities of scales decreased by 8–12%, while specificities increased by 13–16%. Compared with patients with complete data, excluded patients more often had an ischemic (47.9% vs 42.5%) or hemorrhagic (8.7% vs 4.7%) stroke, and had more severe symptoms (median NIHSS 3 (IQR 1–8) vs 1 (IQR 0–3); mean Glasgow Coma Scale score 13.7 (SD 2.5) vs 14.9 (SD 0.65)) (all *p* < 0.01). However, the diagnostic performance of scales relative to each other remained similar (Supplemental Table S4). The sensitivity analysis excluding patients with a TIA (*n* = 470) showed that all scales had an improvement in sensitivity of 4–6% and in NPV of 11–13%, with a slight decrease in PPV (3–4%) (Supplemental Table S5). Relative performances remained consistent. In all analyses, MedPACS had the highest accuracy and NPV, and missed the fewest reperfusion-treated stroke patients. In contrast, LAPSS had the highest specificity and PPV, but the lowest accuracy, sensitivity and NPV, and missed the most stroke patients who received reperfusion treatment.

## Discussion

With our systematic search, we identified 14 prehospital stroke detection scales of which 7 could be reconstructed and used for comparative analysis. Among these, MedPACS, OPSS, and sNIHSS-EMS showed the highest accuracy, sensitivity, and NPV, and missed the fewest reperfusion-treated stroke patients. Conversely, LAPSS had the lowest accuracy, sensitivity, and NPV, and missed the most reperfusion-treated stroke patients. Overall, implementation of a stroke detection scale could reduce unnecessary stroke code activations for patients with a stroke mimic by almost a third. However, all scales failed to detect at least 16% of strokes and, more importantly, more than 10% of reperfusion-treated stroke patients.

The differences between the three best performing scales were marginal. However, whereas the sNIHSS-EMS numerically had the highest sensitivity in all analyses, it also exhibited the lowest specificity, incorporates the most items (six), and has a relatively complex scoring method, which could hamper its use in routine practice. Similarly, although the MedPACS had slightly higher sensitivity and missed fewer reperfusion-treated stroke patients than OPSS, it demonstrated lower specificity and includes all four items of the OPSS with the addition of gaze deviation. Although we did not specifically investigate this, it is important to consider these characteristics and their implications for clinical use when selecting a scale.

The nuanced decision-making process regarding the trade-off between reducing unnecessary stroke code activations accompanied by potentially excessive and costly investigations, and the risk of missing a (reperfusion-treated) stroke patient remains elusive and highly contingent on local resources and logistical circumstances. Consequently, the decision whether to implement a stroke detection scale should be carefully considered based on the context and regional specifics. As expected, the analysis excluding patients with a TIA showed an increase in sensitivity and NPV. Since these patients do not require acute (reperfusion) treatment and thus do not typically necessitate activation of an acute stroke code, this somewhat mitigates the impact of false-negative results from the scales.

Strengths of this study include the comparison of scales in the largest cohort of unselected stroke code patients thus far, and the reconstruction of scales with solely data acquired by paramedics in the field, contributing to its external validity. In addition, the prospective data collection allowed for a comprehensive statistical analysis, and findings of the sensitivity analyses are consistent with our primary results. Furthermore, the proportion of patients diagnosed with a stroke mimic and of reperfusion-treated stroke patients in our cohort corresponds well with findings from other unselected stroke code populations, indicating that our sample is a good reflection of general practice.^5,6^ However, comparison of our results with previous findings is challenging, as in prior studies scales were often retrospectively reconstructed with assessments conducted by ED physicians rather than by EMS paramedics,^10–14,20,22,24,29–32^ stroke codes were selected, excluding, for example, patients with a TIA or stroke mimic,^10,16,21,25^ the study was single-center rather than multicenter or region-based,^9–14,18,20–27,30–33^ or had small sample sizes (e.g. <500 patients).^9,11,14–17,19,21,25–27,32–36^ Consequently, the diagnostic performances of scales reported in previous literature vary widely. For example, whereas one meta-analysis of seven scales found the LAPSS to have the best diagnostic performance,^39^ we found it to have the lowest sensitivity and missed the most reperfusion-treated stroke patients, which is in line with findings of two other studies.^30,38^

Our study has some limitations. First, some stroke patients may have been missed by EMS paramedics. However, from our experience, this happens very infrequently in the Netherlands because paramedics are instructed to activate a stroke code even with the slightest suspicion of a stroke. This is illustrated by the large proportion of patients without any neurological deficit on the NIHSS (28%) and of patients diagnosed with a stroke mimic (33%). Therefore, we feel that this limitation will not substantially influence our findings, especially not the head-to-head comparisons. Second, the FAST scale was already used by EMS paramedics to screen for stroke patients as part of routine care. This may have improved the performance of this scale as well as of the five other scales that include the same clinical items. However, as indicated by the high proportion of FAST-negative patients in both cohorts (approximately 20%), a positive FAST was only one of many triggers for paramedics to activate a stroke code. Hence, we expect any bias introduced by this to have a minimal impact on our results. Third, we adopted the diagnosis and decision whether to administer IVT or EVT from the treating physician, and cannot rule out that some patients with a stroke mimic were misclassified as ischemic stroke or vice versa. However, it is important to note that this pragmatic approach reflects clinical practice. Fourth, some prehospital data were missing for which we used MICE. However, the sensitivity analysis in which missing observations were replaced with findings from corresponding items of the NIHSS at the ED yielded virtually identical results as our primary analysis. Similarly, although in the complete case analysis sensitivities decreased while specificities increased, presumably due to less meticulous documentation of prehospital observations in patients with more severe stroke, the relative diagnostic performances of scales remained essentially the same, confirming the validity of our findings. Fifth, we were not able to compare all identified scales, because available data did not allow for the reconstruction of seven of these. Sixth, we do not have data to investigate the scales’ feasibility, which could be an important feature when considering their implementation. To address these limitations, a new prospective study has been initiated (NCT06332989). Finally, scales were reconstructed and compared with data from current practice in our region. It has to be acknowledged that this practice is context-specific. For example, in the Netherlands, EMS paramedics are registered nurses who generally completed an additional 2-year training in intensive or emergency medical care, and a 1-year training in ambulance care. This could have improved their skills in neurological assessment, and have resulted in an overestimation of diagnostic performance of the studied scales compared to regions with less experienced paramedics. However, it is important to emphasize that this applies to all of the scales and thus will probably not affect the comparison of scale performances substantially.

## Conclusion

This head-to-head comparison of stroke detection scales showed that MedPACS, sNIHSS-EMS, and OPSS had the highest accuracy, sensitivity, and NPV, and missed the fewest reperfusion-treated stroke patients. In contrast, LAPSS performed poorest and missed the most reperfusion-treated stroke patients. Use of the most accurate scale could reduce unnecessary stroke code activations for patients with a stroke mimic by 30%, but at the cost of missing 16% of stroke patients and 10% of ischemic stroke patients who received reperfusion treatment. In practice, the decision whether or not to incorporate a stroke detection scale will be context-specific. Our results provide important background to guide this choice.

## Supplemental Material

sj-docx-1-wso-10.1177_17474930241275123 – Supplemental material for Prehospital stroke detection scales: A head-to-head comparison of 7 scales in patients with suspected stroke Supplemental material, sj-docx-1-wso-10.1177_17474930241275123 for Prehospital stroke detection scales: A head-to-head comparison of 7 scales in patients with suspected stroke by Luuk Dekker, Walid Moudrous, Jasper D Daems, Ewout FH Buist, Esmee Venema, Marcel DJ Durieux, Erik W van Zwet, Els LLM de Schryver, Loet MH Kloos, Karlijn F de Laat, Leo AM Aerden, Diederik WJ Dippel, Henk Kerkhoff, Ido R van den Wijngaard, Marieke JH Wermer, Bob Roozenbeek and Nyika D Kruyt in International Journal of Stroke
