# Supplementary material for: Prehospital stroke detection scales: A head-to-head comparison of 7 scales in patients with suspected stroke
Source: Int J Stroke. 2024 Sep 10;20(3):268–77. doi: 10.1177/17474930241275123 (PMC11874475; doi:10.1177/17474930241275123)
Supplement: sj-docx-1-wso-10.1177_17474930241275123 – Supplemental material for Prehospital stroke detection scales: A head-to-head comparison of 7 scales in patients with suspected stroke [file sj-docx-1-wso-10.1177_17474930241275123.docx]

**SUPPLEMENTAL MATERIAL**

**Supplemental Table S1.** Overview of all identified prehospital stroke detection scales

| Stroke scale | Included scales | | | | | | Excluded scales | | | | | | |
| --- | --- | --- | --- | --- | --- | --- | --- | --- | --- | --- | --- | --- | --- |
|  | **CPSS/**  **FAST**^a^ | **LAPSS** | **MASS** | **OPSS** | **Med**  **PACS** | **sNIHSS-EMS** | **BE-FAST** | **CIVIL-ASAP** | **FPSS** | **GZSS** | **Pre**  **HAST** | **ROSIER** | **SHIPS** |
| Prehospital observation |  |  |  |  |  |  |  |  |  |  |  |  |  |
| Facial droop | 1 | 1 | 1 | 1 | 1 | 0-3 | 1 | - | 1 | 1 | 1 | 1 | - |
| Arm motor function | 1 | 1 | 1 | 1 | 1 | 0-4 | 1 | - | *1*^b^ | 1 | 0-2 | 1 | 0-3^c^ |
| Speech disturbance | 1 | - | 1 | 1 | 1 | 0-3 | 1 | - | 1 | 0.5 | 0-2 | 1 | 2 |
| Grip strength | - | 1 | 1 | - | - | - | - | - | - | - | - | - | - |
| Leg motor function | - | - | - | 1 | 1 | 0-4 | - | - | *1*^b^ | 1 | 0-2 | 1 | 0-3^c^ |
| Gaze deviation | - | - | - | - | 1 | - | - | - | 4 | - | 2 | - | 2 |
| Commands | - | - | - | - | - | - | - | - | - | - | 2 | - | - |
| Sensory deficits | - | - | - | - | - | 0-2 | - | - | - | - | 0-2 | - | - |
| Consciousness | - | - | - | - | - | 0-3 | - | - | - | - | - | - | - |
| Items of excluded scales not prehospitally available |  |  |  |  |  |  |  |  |  |  |  |  |  |
| Visual field defects | - | - | - | - | - | - | 1 | - | 1 | 1 | 2 | 1 | - |
| Gait imbalance | - | - | - | - | - | - | 1 | 1 or -1^d^ | - | - | - | - | - |
| Asymmetry | - | - | - | - | - | - | - | 1 or -1^d^ | - | - | - | - | - |
| Vertigo | - | - | - | - | - | - | - | - | - | -1 | - | - | - |
| Glasgow Coma Scale ≤8 | - | - | - | - | - | - | - | - | - | 2 | - | - | - |
| Loss of consciousness or  syncope | - | - | - | - | - | - | - | - | - | - | - | -1 | - |
| Seizure activity | - | - | - | - | - | - | - | - | - | - | - | -1 | - |
| Stroke risks | - | - | - | - | - | - | - | 1 or -1^d^ | - | - | - | - | - |
| History of seizure  or psychiatry | - | - | - | - | - | - | - | 1 or -1^d^ | - | - | - | - | - |
| History of hypertension | - | - | - | - | - | - | - | - | - | - | - | - | 1 |
| History of atrial fibrillation | - | - | - | - | - | - | - | - | - | - | - | - | 1 |
| Other items of excluded scales |  |  |  |  |  |  |  |  |  |  |  |  |  |
| Age | - | - | - | - | - | - | - | 1 or -1^d^ | - | - | - | - | - |
| Blood glucose | - | - | - | - | - | - | - | -1^d^ | - | - | - | - | - |
| Systolic blood pressure | - | - | - | - | - | - | - | 1 or -1^d^ | - | 1^e^ | - | - | - |
| Diastolic blood pressure | - | - | - | - | - | - | - | - | - | 1^e^ | - | - | - |
| Cutpoint^f^ | **≥1** | **≥1** | **≥1** | **≥1** | **≥1** | **≥1** | **≥1** | **≥1** | **≥1** | **≥2** | **≥1** | **≥1** | **≥3** |

BE-FAST: Balance-Eyes, Face-Arm-Speech-Time test; CIVIL-ASAP, Clinical Information, Vital signs, and Initial Labs – Age, Stroke risks, history of Seizure or psychiatric disease, Sugar level, Asymmetry, not Ambulating, blood Pressure; CPSS indicates Cincinnati Prehospital Stroke Scale; FAST, Face-Arm-Speech-Time test; FPSS, Finnish Prehospital Stroke Scale; GZSS, Guangzhou Stroke Scale; LAPSS, Los Angeles Prehospital Stroke Screen; MASS, Melbourne Ambulance Stroke Screen; MedPACS, Medic Prehospital Assessment for Code Stroke; OPSS, Ontario Prehospital Stroke Screen; PreHAST, PreHospital Ambulance Stroke Test; ROSIER, Recognition Of Stroke In the Emergency Room; SHIPS, Staring-Hypertension-atrIal fibrillation-sPeech-weakneSs; sNIHSS-EMS, shortened National Institutes of Health Stroke Scale for Emergency Medical Services

^a^ CPSS and FAST use the same clinical items, except for a specific sentence to be repeated for CPSS, and were therefore combined.

^b^ FPSS accounts 1 point for limb weakness (either arm and/or leg).

^c^ SHIPS accounts 2 or 3 points in case of unilateral limb weakness

^d^ CIVIL-ASAP uses cut-off values for scoring positive or negative points. *Gait imbalance* is defined as ‘not ambulating’ and is scored +1 if true and -1 if false. *Asymmetry* is defined as ‘any asymmetry’ referring to facial asymmetry or unilateral limb weakness, and is scored +1 if present and -1 if absent. After consultation with the authors of the original study, *stroke risks* is defined as a history of atrial fibrillation and/or myocardial infarction and is scored +1 if present and -1 if absent. *History of seizure or psychiatry* is scored +1 if absent and -1 if present. *Age* is scored +1 if ≥60 years and -1 if ≤40 years. *Blood glucose* is scored -1 if ≤4.4 or ≥22.2 mmol/L (≤70 or ≥400 mg/dL). *Systolic blood pressure* is scored +1 if ≥140 mmHg and -1 if ≤90 mmHg.

^e^ GZSS accounts 1 point for *systolic blood pressure* ≥145 mmHg, and for *diastolic blood pressure* ≥95 mmHg.

^f^ Some scales also aim to more specifically detect ischemic stroke patients with an underlying large-vessel occlusion in the anterior circulation using different cutpoints (FPSS ≥5 points; sNIHSS-EMS ≥6 points; SHIPS ≥6 points).

**Supplemental Table S2.** Overview of missing data

| **Prehospital assessment** | **Number of missing observations**  **(n=3317)** |
| --- | --- |
| Facial droop | 91 (2.7%) |
| Arm motor function | 421 (12.7%) |
| Speech disturbance | 121 (3.6%) |
| Grip strength | 301 (9.1%) |
| Leg motor function | 689 (20.8%) |
| Gaze deviation | 142 (4.3%) |
| Commands | 120 (3.6%) |
| Sensory deficits^a^ | 1797 (54.2%) |
| Consciousness^a^ | 1317 (39.7%) |
| **Total** | **4999 (16.7%)** |

^a^ Data concerning *sensory deficits* and *consciousness* were routinely assessed by paramedics in LPSS, but not in PRESTO.

**Supplemental Table S3.** Diagnostic performance of stroke detection scales after replacing missing prehospital observations with findings from corresponding items of the NIHSS at the ED (n=3016)

| **Stroke scale** | **Accuracy**  **(95% CI)** | **Sensitivity**  **(95% CI)** | **Specificity**  **(95% CI)** | **PPV**  **(95% CI)** | **NPV**  **(95% CI)** | **Proportion of missed reperfusion treatments, % (n)** | | |
| --- | --- | --- | --- | --- | --- | --- | --- | --- |
|  |  |  |  |  |  | **Overall** (n=665) | **IVT**  (n=585) | **EVT**  (n=181) |
| **MedPACS** | 0.67 (0.65-0.69) | 83% (81-84) | 33% (30-36) | 73% (71-75) | 47% (43-50) | 10.8% (72) | 12.0% (70) | 1.1% (2) |
| **OPSS** | 0.67 (0.65-0.69) | 82% (80-84) | 34% (31-37) | 73% (71-75) | 46% (43-50) | 11.7% (78) | 13.0% (76) | 1.7% (3) |
| **sNIHSS-EMS** | 0.67 (0.65-0.69) | 83% (82-85) | 31% (28-34) | 73% (71-74) | 46% (42-50) | 10.8% (72) | 12.0% (70) | 1.7% (3) |
| **MASS** | 0.66 (0.64-0.68) | 81% (79-83) | 33% (30-36) | 72% (71-74) | 44% (40-48) | 13.8% (92) | 15.4% (90) | 1.1% (2) |
| **CPSS/FAST**^a^ | 0.66 (0.64-0.67) | 79% (77-80) | 37% (34-40) | 73% (71-75) | 44% (41-48) | 15.5% (103) | 17.3% (101) | 1.7% (3) |
| **LAPSS** | 0.60 (0.59-0.62) | 65% (63-67) | 51% (48-55) | 74% (72-76) | 40% (37-43) | 26.0% (173) | 28.5% (167) | 6.1% (11) |

301 patients were excluded from this analysis because of a missing grip strength for which no corresponding NIHSS item is available (n=301).

CI: confidence interval; CPSS: Cincinnati Prehospital Stroke Scale; ED: emergency department; EVT: endovascular thrombectomy; FAST: Face-Arm-Speech-Time test; IVT: intravenous thrombolysis; LAPSS: Los Angeles Prehospital Stroke Screen; MASS: Melbourne Ambulance Stroke Screen; MedPACS: Medic Prehospital Assessment for Code Stroke; OPSS: Ontario Prehospital Stroke Screen; PPV: positive predictive value; NIHSS: National Institutes of Health Stroke Scale; NPV: negative predictive value; sNIHSS-EMS: shortened National Institutes of Health Stroke Scale for Emergency Medical Services

^a^ CPSS and FAST use the same clinical items and were therefore combined in the analysis (CPSS/FAST).

**Supplemental Table S4.** Diagnostic performance of prehospital stroke detection scales in complete cases (n=1133)

| **Stroke scale** | **Accuracy**  **(95% CI)** | **Sensitivity**  **(95% CI)** | **Specificity**  **(95% CI)** | **PPV**  **(95% CI)** | **NPV**  **(95% CI)** | **Proportion of missed reperfusion treatments, % (n)** | | |
| --- | --- | --- | --- | --- | --- | --- | --- | --- |
|  |  |  |  |  |  | **Overall** (n=192) | **IVT**  (n=172) | **EVT**  (n=37) |
| **MedPACS** | 0.64 (0.61-0.67) | 75% (72-78) | 45% (40-50) | 70% (67-74) | 51% (46-56) | 17.7% (34) | 18.6% (32) | 5.4% (2) |
| **OPSS** | 0.64 (0.61-0.67) | 75% (71-78) | 46% (41-51) | 71% (67-74) | 51% (46-56) | 18.8% (36) | 19.8% (34) | 5.4% (2) |
| **sNIHSS-EMS** | 0.64 (0.61-0.67) | 76% (72-79) | 44% (39-49) | 70% (67-73) | 51% (45-56) | 18.8% (36) | 19.8% (34) | 5.4% (2) |
| **MASS** | 0.63 (0.60-0.66) | 73% (70-76) | 45% (40-50) | 70% (67-73) | 49% (44-54) | 19.3% (37) | 20.3% (35) | 5.4% (2) |
| **CPSS/FAST**^a^ | 0.63 (0.60-0.66) | 71% (68-75) | 49% (44-54) | 71% (67-74) | 49% (44-54) | 20.8% (40) | 22.1% (38) | 5.4% (2) |
| **LAPSS** | 0.57 (0.54-0.60) | 54% (50-57) | 62% (57-67) | 71% (67-75) | 43% (39-47) | 35.4% (68) | 37.2% (64) | 21.6% (8) |

2184 patients were excluded from this analysis because of one or more missing prehospital observations required for the reconstruction of scales.

CI: confidence interval; CPSS: Cincinnati Prehospital Stroke Scale; EVT: endovascular thrombectomy; FAST: Face-Arm-Speech-Time test; IVT: intravenous thrombolysis; LAPSS: Los Angeles Prehospital Stroke Screen; MASS: Melbourne Ambulance Stroke Screen; MedPACS: Medic Prehospital Assessment for Code Stroke; OPSS: Ontario Prehospital Stroke Screen; PPV: positive predictive value; NPV: negative predictive value; sNIHSS-EMS: shortened National Institutes of Health Stroke Scale for Emergency Medical Services

^a^ CPSS and FAST use the same clinical items and were therefore combined in the analysis (CPSS/FAST).

*Additional information:*

Compared to excluded patients, complete cases were slightly younger (mean 69 [SD 15.2] vs. 72 [SD 14.0] years; *p* < 0.01), had lower pre-stroke morbidity (modified Rankin Scale score 0-2: 91.1% [931/1022] vs. 80.1% [1638/2045]; *p* < 0.01), and were more often primarily presented in a CSC (59.3% [672/1133] vs. 54.8% [1196/2184], *p* = 0.01). Complete cases more often had a stroke mimic (36.5% [413/1133] vs. 30.4% [664/2184]) versus a stroke (63.5% vs. 69.6%: ischemic stroke 42.5% [481/1133] vs. 47.9% [1047/2184]; hemorrhagic stroke 4.7% [53/1133] vs. 8.7% [189/2184]; TIA 16.4% [186/1133] vs. 13.0% [284/2184]; *p* < 0.01) than excluded patients. In addition, they had lower NIHSS scores (median 1 [IQR 0-3] vs. 3 [IQR 1-8]), and less often a decreased consciousness, scoring ‘Alert’ on the Alert/Verbal/Pain/Unresponsive scale more often (96.4% [1092/1133] vs. 72.2% [626/867]), and having higher Glasgow Coma Scale scores (mean 14.9 [SD 0.65], median 15 [IQR 15-15] vs. mean 13.7 (SD 2.5), median 15 [IQR 13-15] (all *p* < 0.01).

**Supplemental Table S5.** Diagnostic performance of prehospital stroke detection scales after excluding patients with a TIA (n=2847)

| **Stroke scale** | **Accuracy**  **(95% CI)** | **Sensitivity**  **(95% CI)** | **Specificity**  **(95% CI)** | **PPV**  **(95% CI)** | **NPV**  **(95% CI)** |
| --- | --- | --- | --- | --- | --- |
| **MedPACS** | 0.66 (0.64-0.68) | 88% (86-89) | 30% (27-33) | 67% (65-69) | 59% (55-64) |
| **OPSS** | 0.66 (0.64-0.67) | 87% (85-88) | 31% (28-34) | 67% (66-69) | 59% (55-63) |
| **sNIHSS-EMS** | 0.65 (0.64-0.67) | 88% (86-90) | 28% (25-31) | 67% (65-69) | 59% (54-63) |
| **CPSS/FAST**^a^ | 0.65 (0.63-0.67) | 84% (82-85) | 34% (31-37) | 68% (66-70) | 56% (52-59) |
| **MASS** | 0.65 (0.63-0.66) | 86% (84-87) | 30% (27-33) | 67% (65-69) | 56% (52-60) |
| **LAPSS** | 0.64 (0.62-0.65) | 72% (70-75) | 49% (46-52) | 70% (68-72) | 52% (49-55) |

470 patients with a TIA were excluded from this analysis.

CI: confidence interval; CPSS: Cincinnati Prehospital Stroke Scale; EVT: endovascular thrombectomy; FAST: Face-Arm-Speech-Time test; IVT: intravenous thrombolysis; LAPSS: Los Angeles Prehospital Stroke Screen; MASS: Melbourne Ambulance Stroke Screen; MedPACS: Medic Prehospital Assessment for Code Stroke; OPSS: Ontario Prehospital Stroke Screen; PPV: positive predictive value; NPV: negative predictive value; sNIHSS-EMS: shortened National Institutes of Health Stroke Scale for Emergency Medical Services; TIA: transient ischemic attack

^a^ CPSS and FAST use the same clinical items and were therefore combined in the analysis (CPSS/FAST).

**Supplemental Figure S1.** PRISMA flowchart of systematic literature search

**Appendix S1.** Search method of systematic literature review

For our literature search, we updated a 2019 Cochrane review on prehospital stroke scales which included publications up to January 2018.^1^ We used this search strategy, which is in line with the strategy of the Cochrane review, to identify new studies assessing stroke detection scales published from January 2016 up to May 2023.

| **PubMed** | ((“Stroke”[majr] OR “Stroke*”[ti] OR “Cerebrovascular Accident*”[ti] OR “CVA”[ti] OR “CVAs”[ti] OR “transient ischemic attack*”[ti] OR “transient ischaemic attack*”[ti] OR “TIA”[ti] OR “TIAs”[ti]) **AND** (“Emergency Medical Services”[Mesh:NoExp] OR “Emergen*”[ti] OR “prehospital”[ti] OR “prehospital”[ti] OR “EMS”[ti] OR “Emergencies”[majr] OR “ambulance*”[ti] OR “Emergency Medical Technicians”[majr]) **AND** (“Triage”[majr] OR “triag*”[ti] OR “Early Diagnosis”[majr] OR “recogni*”[ti] OR “scale*”[ti] OR “screen*”[ti] OR “identif*”[ti] OR “diagnos*”[ti] OR “predict*”[ti])) OR (“Cincinnati Prehospital Stroke Scale”[tw] OR “Los Angeles Prehospital Stroke Scale”[tw] OR “Los Angeles Prehospital Stroke Scale”[tw] OR “Melbourne Ambulance Stroke”[tw] OR “Ontario Prehospital Stroke Screening Tool”[tw] OR “OPSST”[tiab] OR “Face Arm Speech Time”[tw] OR “Recognition of Stroke in the Emergency Room”[tw] OR “Recognition of Stroke in the Emergency Department”[tw] OR “ROSIER”[tiab] OR “Medic Pre-hospital Assessment for Code Stroke”[tw] OR “Medic Prehospital Assessment for Code Stroke”[tw] OR “Med PACS”[tiab] OR “PreHospital Ambulance Stroke Test”[tw] OR “Pre-hospital Ambulance Stroke Test”[tw] OR “PreHAST”[tiab]) **AND** (“2016/01/01”[Date – Publication] : “2023/05/01”[Date – Publication]) |
| --- | --- |
| **EMBASE** | ((((exp *cerebrovascular accident/ or “Cerebrovascular Accident*”.ti. or “Stroke*”.ti. or “CVA”.ti. or “CVAs”.ti. or “transient ischemic attack*”.ti. or “transient ischaemic attack*”.ti. or “TIA”.ti. or “TIAs”.ti.) **and** (*emergency health service/ or “Emergen*”.ti. or “prehospital”.ti. or “prehospital”.ti. or “EMS”.ti. or “Emergency”/ or “ambulance*”.ti. or exp *ambulance/ or exp *rescue personnel/) **and** (“triag*”.ti. or exp *early diagnosis/ or “recogni*”.ti. or “scale*”.ti. or “screen*”.ti. or “identif*”.ti. or “diagnos*”.ti. or “predict*”.ti.)) or ((“Cincinnati Prehospital Stroke Scale” or “Los Angeles Prehospital Stroke Scale” or “Los Angeles Pre-hospital Stroke Scale” or “Melbourne Ambulance Stroke” or “Ontario Prehospital Stroke Screening” or “Face Arm Speech Time” or “Recognition of Stroke in the Emergency Room” or “Recognition of Stroke in the Emergency Department”).mp. or “ROSIER”.ti,ab. Or “Medic Prehospital Assessment for Code Stroke”.mp. or “Medic Prehospital Assessment for Code Stroke”.mp. or “Med PACS”.ti,ab. Or “PreHospital Ambulance Stroke Test”.mp. or “Pre-hospital Ambulance Stroke Test”.mp. or “PreHAST”.ti,ab.)) **and** 20160101:20230501.(sa_year).) not (conference or conference abstract or “conference review”).pt. |

**Appendix S2.** Specification of imputation method with MICE

Imputation was performed in R (version 4.1.2) using the MICE package (version: 3.14.0).^2,3^ All patients from the pooled LPSS/PRESTO dataset were included in the imputation dataset (n=3321). The following variables were manually selected and included as predictors in the imputation model: age; sex; pre-stroke modified Rankin Scale score; history of: atrial fibrillation, diabetes mellitus, hypertension, hypercholesterolemia, intracranial hemorrhage, myocardial infarction, and peripheral arterial disease; wake-up stroke (yes/no); prehospital systolic blood pressure; prehospital diastolic blood pressure; prehospital assessment of: questions, commands, abnormal speech, gaze deviation, facial palsy, grip strength, arm motor function, leg motor function, agnosia, sensory deficits, tactile extinction, and consciousness as measured with the Alert/Verbal/Pain/Unresponsive (AVPU) score; onset-to-door time; systolic blood pressure at the ED; diastolic blood pressure at the ED; blood glucose level at the ED; assessment of all 11 NIHSS items at the ED separately; final diagnosis (after adjudication by the imaging core-lab for PRESTO); and study (LPSS or PRESTO). The MICE function quickpred() was used to specify the predictor matrix of the imputation model. The function’s default settings were used with age, sex and final diagnosis set to always be included as predictors for the incomplete target variables. Furthermore, study was not used as a predictor for the following four variables that were not routinely documented in all patients in PRESTO: prehospital assessment of sensory deficits, tactile extinction, and consciousness as measured with the Alert/Verbal/Pain/Unresponsive (AVPU) score (not documented in PRESTO); and prehospital assessment of agnosia (only documented in patients with left-sided hemiparesis in PRESTO). The imputation methods of incomplete target variables were set by MICE and based on the type of variable (e.g. numeric, ordinal, nominal). A total of 5 imputation datasets were created with 20 iterations. There were no logged events, and the convergence trace lines intermingled well and showed no trend. Lastly, after imputation, the exclusion criteria (i.e. age ≥18 years and available hospital records) were applied, resulting in the analyzed dataset.

**APPENDICES REFERENCES**

1. Zhelev Z, Walker G, Henschke N, Fridhandler J, Yip S. Prehospital stroke scales as screening tools for early identification of stroke and transient ischemic attack. *Cochrane Database Syst Rev* 2019; 4(4):CD011427. DOI: 10.1002/14651858.CD011427.pub2.

2. R Core Team. R: A language and environment for statistical computing. R Foundation for Statistical Computing, Vienna, Austria, 2021. https://www.R-project.org/

3. van Buuren S, Groothuis-Oudshoorn K. mice: Multivariate Imputation by Chained Equations in R. *J Stat Soft* 2011; 45(3): 1–67. DOI: 10.18637/jss.v045.i03.
